# Evolution of seed mass associated with mating systems in multiple plant families

**DOI:** 10.1111/jeb.13949

**Published:** 2021-10-26

**Authors:** Hirofumi Tateyama, Kaori Chimura, Takashi Tsuchimatsu

**Affiliations:** ^1^ Department of Biological Sciences Graduate School of Science The University of Tokyo Tokyo Japan; ^2^ Graduate School of Science and Technology Chiba University Chiba Japan

**Keywords:** mating systems, parent–offspring conflict, seed mass, selfing, selfing syndrome

## Abstract

In flowering plants, the evolution of self‐fertilization (selfing) from obligate outcrossing is regarded as one of the most prevalent evolutionary transitions. The evolution of selfing is often accompanied by various changes in genomic, physiological and morphological properties. In particular, a set of reproductive traits observed typically in selfing species is called the “selfing syndrome”. A mathematical model based on the kinship theory of genetic imprinting predicted that seed mass should become smaller in selfing species compared with outcrossing congeners, as a consequence of the reduced conflict between maternally and paternally derived alleles in selfing plants. Here, we test this prediction by examining the association between mating system and seed mass across a wide range of taxa (642 species), considering potential confounding factors: phylogenetic relationships and growth form. We focused on three plant families—Solanaceae, Brassicaceae and Asteraceae—where information on mating systems is abundant, and the analysis was performed for each family separately. When phylogenetic relationships were controlled, we consistently observed that selfers (represented by self‐compatible species) tended to have a smaller seed mass compared with outcrossers (represented by self‐incompatible species) in these families. In summary, our analysis suggests that small seeds should also be considered a hallmark of the selfing syndrome, although we note that mating systems have relatively small effects on seed mass variation.

## Graphical abstract

Association between seed mass and mating systems (self‐incompatible [SI] and self‐compatible [SC]) in three plant families.

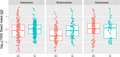

## 1 INTRODUCTION

In flowering plants, the evolution of self‐fertilization (selfing) from obligate outcrossing is regarded as one of the most prevalent evolutionary transitions (Barrett, 2002; Goldberg et al., 2010; Shimizu & Tsuchimatsu, 2015; Stebbins, 1974). The evolution of selfing is reported to have profound effects on the various aspects of genomic, physiological and morphological properties, such as nucleotide diversity, effective population size, the extent of linkage disequilibrium, geographical distribution, gamete numbers and morphology of reproductive traits (Barrett, 2002; Brandvain et al., 2014; Cruden, 2000; Grossenbacher et al., 2017; Shimizu & Tsuchimatsu, 2015; Sicard & Lenhard, 2011) In particular, a set of reproductive traits observed typically in selfing species is called the “selfing syndrome”, including small flower size, loss of herkogamy and reduced pollen number. The molecular and genetic bases for the evolution of the selfing syndrome have been studied extensively: for example, reduction in petal size (Sicard & Lenhard, 2011; Woźniak et al., 2020) and in pollen number (Tsuchimatsu et al., 2020).

Seed mass is a key trait that is a central to many aspects of plant ecology, such as viability, dispersal, and seedling growth (Baskin & Baskin, 2014; Moles et al., 2005; Stanton, 1984). Given that there is a trade‐off between seed number and seed mass (Smith & Fretwell, 1974), De Jong et al. (2005) proposed a mathematical model predicting that seed mass variation would also be influenced by the evolution of selfing. This model stems from Haig's kinship theory of genetic imprinting (Haig, 1997), which postulated that optimal seed mass differs for mothers and for offspring, given the conflict of interests between maternally and paternally derived alleles. Because the degree of conflict should depend on the selfing rate of maternal plants, De Jong et al. (2005) predicted that outcrossing plant species tend to produce larger seeds compared with selfers. They tested this prediction using data from 265 common British grassland plants (Grime et al., 1988) and found that the mean seed mass of selfers was smaller than that of outcrossers. Although this was consistent with what the model predicted, the test was a simple comparison of mean seed mass, and this was potentially confounded by phylogeny or growth form that are interrelated with breeding systems.

Here we tested the prediction of De Jong et al. (2005) more formally, using a mixed model‐based regression analysis. We explicitly considered the following factors. First, we took phylogenetic confounding into account. We focused on three plant families, Solanaceae, Brassicaceae and Asteraceae, where information on mating systems is available for hundreds of species, and the analysis was performed for each family separately. We considered the genus‐level phylogenetic information as a random effect in the framework of Bayesian linear mixed model (the details are presented in the Materials and Methods). Second, we took growth forms into consideration, which suggested to be most strongly associated with seed mass (Moles et al., 2005). Whilst most species are herbaceous in these three studied families, non‐herbaceous plants (trees, vines or shrubs) are also common in the Solanaceae (Table 1). We included this factor as a fixed effect in the mixed model, which allowed us to quantify the relative contributions to seed mass variation. Through this analysis, we generally found that selfers (represented by self‐compatible species) tended to have smaller seed mass compared with outcrossers (represented by self‐incompatible species) in the three families studied, although the result varied by plant families and methods. Recently, Mazer et al. (2020) also reported that considering climatic variables, seed mass was correlated with the mating system in the genus *Clarkia*. Our study, using 642 species from three families complements that of Mazer et al. (2020) and further supports the correlation between seed mass and mating systems in a wider range of plant taxa.

**TABLE 1:** The numbers of genera and species used in this study

	Asteraceae	Brassicaceae	Solanaceae
Number of genera	117	56	15
Number of species	345	153	144
Number of self‐incompatible species	209	58	37
Number of self‐compatible species	136	95	107
Number of herbaceous speces	325	151	97
Number of non‐herbaceous speces	20	2	47

## 2 MATERIALS AND METHODS

### 2.1 Data collection

In this study, we focused on three plant families, Solanaceae, Brassicaceae and Asteraceae, because their mating systems have been studied extensively and information is available for hundreds of species in these families (*e*.*g*. Goldberg et al., 2010; Grossenbacher et al., 2017). We first collected information on the mating systems of the species. As a proxy for selfing rate, we used information on self‐incompatibility (SI) and self‐compatibility (SC), because the available data of selfing rate was limited compared with the accumulated knowledge of SI and SC. We exploited the data of two papers (Goldberg et al., 2010 for Solanaceae, and Grossenbacher et al., 2017 for Solanaceae, Brassicaceae and Asteraceae) and also collected data by searching the literature manually, especially for genera in which mating system evolution is well studied (e.g. *Petunia* and *Brassica*). Goldberg et al. (2010) classified the mating system as 0, SI; 1, SC; 2, SI + SC; 3, SI + SC + Dioecy; 4, Dioecy; and 5, SI + Dioecy. In this study, we only used the species classified as 0 (SI) or 1 (SC). We then collected the seed mass data of the species in which we obtained the data of mating systems, mostly from Kew's Seed Information Database (SID http://data.kew.org/sid/). The SID describes a mean value of 1000 seed mass for each species when multiple data are available; here, we used mean values for analysis.

To deal with confounding by growth form, we obtained information on whether the species are herbaceous, trees, vines or shrubs. These data were mostly obtained from Engemann et al. (2016) and Global Biodiversity Information Facility (GBIF; https://www.gbif.org/), but when they were not available, we searched the literature directly.

We obtained the genus‐level phylogenetic information to be incorporated as a random factor in Bayesian linear mixed model (MCMCglmm). We used the Phylomatic platform (Webb & Donoghue, 2005) with the dataset of 32 223 plant species reported by Zanne et al. (2014). This dataset did not cover all the species but almost all the genera for which we obtained the phenotypic data. We, therefore, first generated a genus‐level phylogenetic tree for each family, by randomly choosing one available species per genus (Figure S1). Then, we included all the used species in the tree, assuming the same phylogenetic distance (0.000001) within the genus. We excluded genera in which phylogenetic data were not available for any species.

**FIGURE 1:**
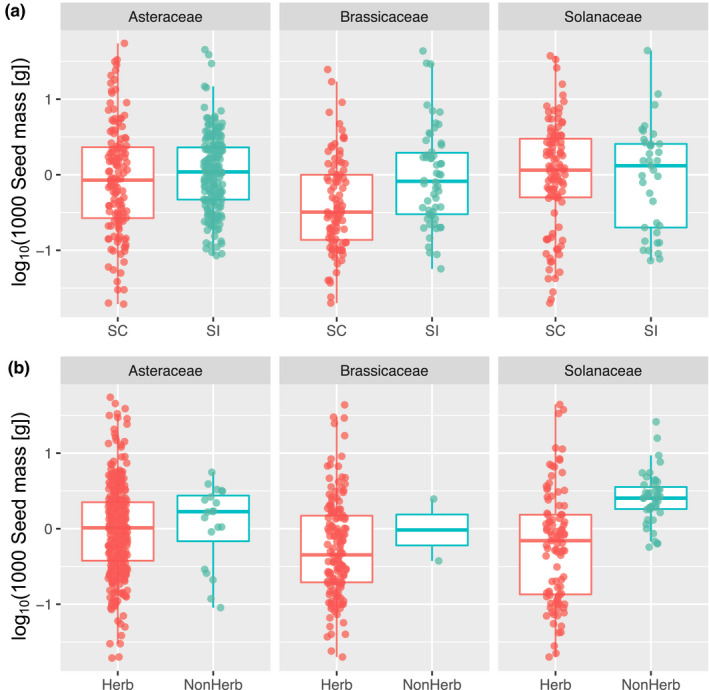
Seed mass [log_10_(1000 seed mass [g])] variation in the Asteraceae, Brassicaceae and Solanaceae. (a) Relationship between seed mass and mating systems (self‐incompatible [SI] and self‐compatible [SC]). (b) Relationship between seed mass and growth forms (herbaceous [Herb] and non‐herbaceous [NonHerb]). (a, b) Boxplots show centre line: median; box limits: upper and lower quartiles; whiskers: not >1.5 times the interquartile range

In total, we used 345 species (117 genera) for Asteraceae, 153 species (56 genera) for Brassicaceae and 144 species (15 genera) for Solanaceae (Table 1). An overview of the relationship between seed mass, mating systems, and growth forms is summarized in Figure 1. The numbers of SI and SC species and of herbaceous species and non‐herbaceous (trees, vines or shrubs) for each family used in this study are shown in Table 1. All the data including references are available in Table S1.

### 2.2 Data analysis

All the data analysis was performed by using R version 3.6.3 (R Core Team, 2020). For each family, we first performed an analysis without phylogenetic information: a simple analysis of variance (ANOVA) for each family, in which mating systems (SI/SC) and growth forms were the explanatory variables, and log_10_(1000 seed mass [g]) was the response variable, by using lm and anova functions. We also performed a regression analysis in which genera were the fixed effect to quantify the between‐genera variance of seed mass.

To deal with the confounding by phylogeny, we performed Bayesian linear mixed model by using the R library MCMCglmm (Hadfield, 2010), in which mating systems (SI/SC) and growth forms were the explanatory variables, and log_10_(1000 seed mass [g]) was the response variable. Growth forms were classified into two categories: herbaceous or non‐herbaceous (tree, vine or shrub) (Table 1). We included the phylogenetic information as a random effect. We specified priors *V* = 1 and nu = 0.02 for R and G structures, and a Gaussian distribution was assumed. We ran 1 000 000 iterations with a burn‐in of 1000 iterations and a thinning interval of 1 (no thinning, as recommended by Link and Eaton (2012)). The MCMC simulations generally generated well‐converged posterior distributions. The trees generated from the Phylomatic platform were mostly ultrametric, but we slightly corrected by the force.ultrametric function of phytools to make usable for MCMCglmm (Revell, 2012). The used The R script, phenotypic data, and phylogenetic trees in newick format are available at GitHub (https://github.com/tsuchimatsu/seed_mass).

## 3 RESULTS

We first performed ANOVA with mating systems (SI/SC) and growth forms as the explanatory variable, seed mass as the response variable. We found that the trend varied between plant families: Seed mass tended to be smaller in SC species compared with SI species for the Brassicaceae but not for the Asteraceae and the Solanaceae (*p* = 0.0009138 for Brassicaceae, *p* = 0.1426 for Asteraceae and *p* = 0.7746 for Solanaceae; Table 2). *R* ^2^ values were low in all three families (*R* ^2^ = 0.006272 for Asteraceae; *R* ^2^ = 0.0709 for Brassicaceae; *R* ^2^ = 0.00048 for Solanaceae), suggesting that, even if significant, mating systems have relatively small effects on seed mass variation. The effect of growth forms was insignificant in the Asteraceae and the Brassicaceae, possibly due to the limited number of non‐herbaceous species, but the effect was highly significant in the Solanaceae where non‐herbaceous species are abundant (*p* = 2.078 × 10^−7^). A regression analysis with genera as the explanatory variable and seed mass as the response variable revealed that the large portions of seed mass variation are the between‐genera variance (*R* ^2^ = 0.8473 for Asteraceae; *R* ^2^ = 0.7576 for Brassicaceae; *R* ^2^ = 0.8203 for Solanaceae).

**TABLE 2:** Summary of an analysis of variance for mating systems and growth forms

	*df*	Sum Sq	Mean Sq	*F* value	Pr (>*F*)
Asteraceae					
Mating system	1	0.775	0.7750	2.1592	0.1426
Growth form	1	0.039	0.03927	0.1094	0.741
Residuals		122.76	0.3590		
Brassicaceae					
Mating system	1	4.551	4.5508	11.4467	0.0009138
Growth form	1	0.001	0.0008	0.0019	0.9650
Residuals	150	59.634	0.3976		
Solanaceae					
Mating system	1	0.034	0.0341	0.0823	0.7746
Growth form	1	12.353	12.3532	29.8234	2.08E‐07
Residuals	141	58.404	0.4142		

We then performed Bayesian linear mixed model by using MCMCglmm (Table 3). We generally found that mating systems have significant effects on seed mass variation (*P*_MCMC_ = 0.040 for Asteraceae; *P*_MCMC_ = 0.0405 for Brassicaceae; *P*_MCMC_ = 0.00656 for Solanaceae). In all three families, the mean values of the effect of mating systems were negative, suggesting that seed mass tended to be smaller in SC species compared with SI species (−0.09819 [95% Credible Interval (CI): −0.1921, −0.005127] for Asteraceae; −0.2006 [95% CI: −0.3936, −0.01140] for Brassicaceae; −0.2320 [95% CI: −0.3974, −0.06634] for Solanaceae). The effect of growth forms was insignificant in the Asteraceae (*P*_MCMC_ = 0.743) and the Brassicaceae (*P*_MCMC_ = 0.6962), possibly due to the limited number of non‐herbaceous species, but the effect was highly significant in the Solanaceae where non‐herbaceous species are abundant (*P*_MCMC_ = 0.00191). In the Solanaceae, the mean value of the effect of growth forms was positive, suggesting that seed mass tended to be larger in non‐herbaceous species compared with herbaceous species (0.2314 [95% CI: 0.08608, 0.3749]).

**TABLE 3:** Summary of Bayesian linear mixed model with phylogenetic information

	Mean of posterior distribution	95% Credible interval	*P*_MCMC_
Asteraceae			
Mating system	−0.09819	[−0.1921, −0.005127]	0.040
Growth form	0.03769	[−0.1888, 0.2638]	0.743
Brassicaceae			
Mating system	−0.2006	[−0.3936, −0.01140]	0.0405
Growth form	−0.1334	[−0.8153, 0.5431]	0.6962
Solanaceae			
Mating system	−0.2320	[−0.3974, −0.06634]	0.00656
Growth form	0.2314	[0.08608, 0.3749]	0.00191

## 4 DISCUSSION

We performed a meta‐analysis on the relationship between seed mass and mating systems in three plant families (Solanaceae, Brassicaceae and Asteraceae), by explicitly taking potential confounding factors into account: phylogenetic relationships and growth forms. We found that SC species generally show smaller seed mass compared with their SI congeners (Figure 1; Table 3). Whilst we obtained mixed support in ANOVA without the information of phylogeny (Table 2), we consistently detected significant effects of mating systems in the Bayesian linear mixed model, in which phylogenetic relationships were controlled as a random factor.

Several previous studies have reported that self‐fertilizing taxa produce smaller seeds than their outcrossing congeners in specific taxa (*e*.*g*. Knies et al., 2004; Mazer et al., 2020; Mitchell‐Olds, 2001; Sharma et al., 1999). Mazer et al. (2020) reported the correlation between mating system and seed mass by controlling for potential confounding factors such as climate variables in *Clarkia*. Our study also found correlations between seed mass and mating system in a much wider range of plant taxa by controlling for possible confounding factors, thereby serving as a complementary study to Mazer et al. (2020). Our results support the emerging notion that small seed mass also constitutes a component of the selfing syndrome (Mazer et al., 2020; Ornduff, 1969; Shimizu & Tsuchimatsu, 2015; Sicard & Lenhard, 2011). However, our caveat is that, although the effect of mating systems was significant, its effect on seed mass variation was relatively small. We rather found that the large portions of seed mass variation were due to the between‐genera variance. Given that the evolutionary transition from SI to SC occurs frequently within each genus (Goldberg et al., 2010; Igic et al., 2006; Shimizu & Tsuchimatsu, 2015), the portion of seed mass variation explained by mating systems would be relatively limited.

Nonetheless, our results are consistent with the theoretical model (De Jong et al., 2005), which predicted that outcrossing plant species tend to produce larger seeds compared with selfers according to Haig's kinship theory of genetic imprinting (Haig, 1997). However, we note that the correlation between mating system and seed mass might have other causes. First, selfing would be expected to coevolve with smaller seed mass because both attributes might increase the ability for colonization (Mazer et al., 2020). This is because selfing plants can reproduce without mates or pollinators (Baker, 1955; Darwin, 1876), and smaller seeds are expected to disperse farther than larger ones (Greene & Johnson, 1993; Tamme et al., 2014). Second, sex allocation theory predicts that the pollen‐ovule (P/O) ratio should increase linearly with increasing seed mass amongst seeding plants (Charnov, 1986; Götzenberger et al., 2006). Götzenberger et al. (2006) indeed found a positive correlation between the P/O ratio and seed mass through a meta‐analysis. Because the P/O ratio tends to be lower in selfing species (Cruden, 2000), the correlation between mating system and seed mass could have arisen as a consequence. These hypotheses are not mutually exclusive; thus, it is possible that these effects might have jointly led to the observed correlation between mating system and seed mass. Further analysis including these factors as covariates may help quantify the direct effect of mating systems. Willi (2013) tested the effect of parental conflict on seed mass variation by diallel crosses in *Arabidopsis lyrata*. That study showed that seeds were larger when pollen came from another outcrossing population than when pollen came from a selfing or the same population, providing support for the idea of parental conflict between male‐derived selfish genes and female recognition genes. Cailleau et al. (2018) also tested the effect of parent‐offspring conflict towards resource allocation in seed development through diallel crosses in maize. Raunsgard et al. (2018) found that more outcrossed paternal populations produce larger seeds when crossed with less outcrossed maternal populations—and vice versa—in *Dalechampia scandens*. Given these findings, it is possible that mating systems, which affect the degree of parental conflict, might have important roles in determining seed mass variation across species.

We acknowledge that polyploidy may also be a confounding factor in this analysis. Polyploidization is often accompanied by evolution of SC and self‐fertilization (e.g. Barringer, 2007; Entani et al., 1999; Lewis, 1947; Miller & Venable, 2000; Novikova et al., 2017; Robertson et al., 2011; Stebbins, 1950; Tsuchimatsu et al., 2012). Thus, the association between seed mass and mating systems may partly be influenced by polyploidy, but it would rather have an opposite effect, given that polyploid species tend to produce larger seeds (e.g. Bretagnolle et al., 1995; Eliášová & Münzbergová, 2014; Miller et al., 2012; Stevens et al., 2020). Whilst we did not distinguish diploid and polyploid species in this study, the association between seed mass and mating systems may even become stronger when the ploidy level is controlled. We note, however, that the mechanism of self‐incompatibility varies between families, which also makes the effect of polyploidization on mating system transition different. Specifically, whereas polyploidization almost always disrupt SI in gametophytic SI systems, in sporophytic SI systems, polyploidization does not necessarily induce the loss of SI (e.g. Mable et al., 2004). It would be important to extend the analysis to a broader scale beyond three studied families.

In summary, we found evidence that selfing species tend to produce smaller seeds after controlling for possible confounding factors in multiple plant families, albeit its relatively small effect. This suggests that small seeds could also be considered a hallmark of the selfing syndrome, which was previously not well‐recognized (Mazer et al., 2020). Whilst several traits have been reported as components of the selfing syndrome, thanks to the increase in accumulated phenotypic and genomic data in many taxa, more traits may found to be typical features of selfing species (Ornduff, 1969; Shimizu & Tsuchimatsu, 2015; Sicard & Lenhard, 2011).

## CONFLICT OF INTEREST

The authors have no conflict of interest to declare.

## AUTHOR CONTRIBUTIONS

TT conceived and designed the research. HT, KC and TT collected the data. HT and TT performed the analysis. HT and TT wrote the paper.

### PEER REVIEW

The peer review history for this article is available at https://publons.com/publon/10.1111/jeb.13949.

## Supporting information

Fig S1

Table S1

## Data Availability

All the data and scripts are available at GitHub (https://github.com/tsuchimatsu/seed_mass).

## References

[jeb13949-bib-0001] Baker, H. G. (1955). Self‐compatibility and establishment after “long‐distance” dispersal. Evolution, 9, 347–349. 10.2307/2405656

[jeb13949-bib-0002] Barrett, S. C. H. (2002). The evolution of plant sexual diversity. Nature Reviews Genetics, 3, 274–284. 10.1038/nrg776 11967552

[jeb13949-bib-0003] Barringer, B. C. (2007). Polyploidy and self‐fertilization in flowering plants. American Journal of Botany, 94, 1527–1533. 10.3732/ajb.94.9.1527 21636519

[jeb13949-bib-0004] Baskin, C. C. , & Baskin, J. (2014). Seeds. 2nd ed. Academic Press.

[jeb13949-bib-0005] Brandvain, Y. , Kenney, A. M. , Flagel, L. , Coop, G. , & Sweigart, A. L. (2014). Speciation and Introgression between *Mimulus nasutus* and *Mimulus guttatus* . PLoS Genetics, 10. e1004410. 10.1371/journal.pgen.1004410 24967630 PMC4072524

[jeb13949-bib-0006] Bretagnolle, F. , Thompson, J. D. , & Lumaret, R. (1995). The influence of seed size variation on seed germination and seedling vigour in diploid and tetraploid *Dactylis glomerata* L. Annals of Botany, 76, 607–615. 10.1006/anbo.1995.1138

[jeb13949-bib-0007] Cailleau, A. , Grimanelli, D. , Blanchet, E. , Cheptou, P.‐O. , & Lenormand, T. (2018). Dividing a maternal pie among half‐sibs: Genetic conflicts and the control of resource allocation to seeds in maize. The American Naturalist, 192, 577–592. 10.1086/699653 30332585

[jeb13949-bib-0008] Charnov, E. L. (1986). An optimisation principle for sex allocation in a temporally varying environment. Heredity, 56, 119–121. 10.1038/hdy.1986.16

[jeb13949-bib-0009] Cruden, R. W. (2000). Pollen grains: why so many? Plant Systematics and Evolution, 222, 143–165. 10.1007/978-3-7091-6306-1_8

[jeb13949-bib-0010] Darwin, C. R. (1876). The effects of cross and self fertilisation in the vegetable kingdom. John Murray.

[jeb13949-bib-0011] De Jong, T. J. , Van Dijk, H. , & Klinkhamer, P. G. L. (2005). Hamilton’s rule, imprinting and parent‐offspring conflict over seed mass in partially selfing plants. Journal of Evolutionary Biology, 18, 676–682. 10.1111/j.1420-9101.2004.00856.x 15842497

[jeb13949-bib-0012] Eliášová, A. , & Münzbergová, Z. (2014). Higher seed size and germination rate may favour autotetraploids of *Vicia cracca* L. (Fabaceae). Biological Journal of the Linnean Society, 113, 57–73. 10.1111/bij.12318

[jeb13949-bib-0013] Engemann, K. , Sandel, B. , Boyle, B. , Enquist, B. J. , Jørgensen, P. M. , Kattge, J. , McGill, B. J. , Morueta‐Holme, N. , Peet, R. K. , Spencer, N. J. , Violle, C. , Wiser, S. K. , & Svenning, J.‐C. (2016). A plant growth form dataset for the New World. Ecology, 97, 3243. 10.1002/ecy.1569 27870054

[jeb13949-bib-0014] Entani, T. , Takayama, S. , Iwano, M. , Shiba, H. , Che, F.‐S. , & Isogai, A. (1999). Relationship between polyploidy and pollen self‐incompatibility phenotype in *Petunia hybrida* vilm. Bioscience, Biotechnology, and Biochemistry, 63, 1882–1888. 10.1271/bbb.63.1882 10635553

[jeb13949-bib-0015] Goldberg, E. E. , Kohn, J. R. , Lande, R. , Robertson, K. A. , Smith, S. A. , & Igić, B. (2010). Species selection maintains self‐incompatibility. Science, 330, 493–495. 10.1126/science.1194513 20966249

[jeb13949-bib-0016] Götzenberger, L. , Durka, W. , Kühn, I. , & Klotz, S. (2006). The relationship between the pollen‐ovule ratio and seed size: A comparative test of a sex allocation hypothesis. Evolutionary Ecology Research, 8, 1101–1116.

[jeb13949-bib-0017] Greene, D. F. , & Johnson, E. A. (1993). Seed mass and dispersal capacity in wind‐dispersed diaspores. Oikos, 67, 69–74. 10.2307/3545096

[jeb13949-bib-0018] Grime, J. P. , Hodgson, J. G. , & Hunt, R. (1988). Comparative plant ecology: A functional approach to common British species. Springer Netherlands. 10.1007/978-94-017-1094-7

[jeb13949-bib-0019] Grossenbacher, D. L. , Brandvain, Y. , Auld, J. R. , Burd, M. , Cheptou, P. O. , Conner, J. K. , Grant, A. G. , Hovick, S. M. , Pannell, J. R. , Pauw, A. , Petanidou, T. , Randle, A. M. , Rubio de Casas, R. , Vamosi, J. , Winn, A. , Igic, B. , Busch, J. W. , Kalisz, S. , & Goldberg, E. E. (2017). Self‐compatibility is over‐represented on islands. New Phytologist, 215, 469–478. 10.1111/nph.14534 28382619

[jeb13949-bib-0020] Hadfield, J. D. (2010). MCMC methods for multi‐response generalized linear mixed models: The MCMCglmm R package. Journal of Statistical Software, 33, 1–22. 10.18637/jss.v033.i02 20808728 PMC2929880

[jeb13949-bib-0021] Haig, D. (1997). Parental antagonism, relatedness asymmetries, and genomic imprinting. Proceedings of the Royal Society B: Biological Sciences, 264, 1657–1662. 10.1098/rspb.1997.0230 PMC1688715 9404029

[jeb13949-bib-0022] Igic, B. , Bohs, L. , & Kohn, J. R. (2006). Ancient polymorphism reveals unidirectional breeding system shifts. Proceedings of the National Academy of Sciences of the United States of America, 103, 1359–1363. 10.1073/pnas.0506283103 16428289 PMC1360522

[jeb13949-bib-0023] Knies, J. L. , Delesalle, V. A. , & Cavaliere, A. R. (2004). Seed mass and morphology in outcrossing and selfing species of *Clarkia* (Onagraceae): An sem study. International Journal of Plant Sciences, 165, 85–96. 10.1086/380979

[jeb13949-bib-0025] Lewis, D. (1947). Competition and dominance of incompatibility alleles in diploid pollen. Heredity, 1, 85–108. 10.1038/hdy.1947.5

[jeb13949-bib-0026] Link, W. A. , & Eaton, M. J. (2012). On thinning of chains in MCMC. Methods in Ecology and Evolution, 3, 112–115. 10.1111/j.2041-210X.2011.00131.x

[jeb13949-bib-0027] Mable, B. K. , Beland, J. , & Di Berardo, C. (2004). Inheritance and dominance of self‐incompatibility alleles in polyploid *Arabidopsis lyrata* . Heredity, 93, 476–486. 10.1038/sj.hdy.6800526 15266298

[jeb13949-bib-0028] Mazer, S. J. , Park, I. M. , Kimura, M. , Maul, E. M. , Yim, A. M. , & Peach, K. (2020). Mating system and historical climate conditions affect population mean seed mass: Evidence for adaptation and a new component of the selfing syndrome in *Clarkia* . Journal of Ecology, 108, 1523–1539. 10.1111/1365-2745.13338

[jeb13949-bib-0029] Miller, J. S. , & Venable, D. L. (2000). Polyploidy and the evolution of gender dimorphism in plants. Science, 289, 2335–2338. 10.1126/science.289.5488.2335 11009416

[jeb13949-bib-0030] Miller, M. , Zhang, C. , & Chen, Z. J. (2012). Ploidy and hybridity effects on growth vigor and gene expression in *Arabidopsis thaliana* hybrids and their parents. G3 Genes|Genomes|Genetics, 2, 505–513. 10.1534/g3.112.002162 22540042 PMC3337479

[jeb13949-bib-0031] Mitchell‐Olds, T. (2001). *Arabidopsis thaliana* and its wild relatives: A model system for ecology and evolution. Trends in Ecology and Evolution, 16, 693–700. 10.1016/S0169-5347(01)02291-1

[jeb13949-bib-0032] Moles, A. T. , Ackerly, D. D. , Webb, C. O. , Tweddle, J. C. , Dickie, J. B. , Pitman, A. J. , & Westoby, M. (2005). Factors that shape seed mass evolution. Proceedings of the National Academy of Sciences of the United States of America, 102, 10540–10544. 10.1073/pnas.0501473102 16030149 PMC1180762

[jeb13949-bib-0033] Novikova, P. Y. , Tsuchimatsu, T. , Simon, S. , Nizhynska, V. , Voronin, V. , Burns, R. , Fedorenko, O. M. , Holm, S. , Säll, T. , Prat, E. , Marande, W. , Castric, V. , & Nordborg, M. (2017). Genome sequencing reveals the origin of the Allotetraploid *Arabidopsis suecica* . Molecular Biology and Evolution, 34, 957–968. 10.1093/molbev/msw299 28087777 PMC5400380

[jeb13949-bib-0034] Ornduff, R. (1969). Reproductive biology in relation to systematics. Taxon, 18, 121–133. 10.2307/1218671

[jeb13949-bib-0035] R Core Team (2020). R: A language and environment for statistical computing. R Foundation for Statistical Computing. Retrieved from https://www.R‐project.org/

[jeb13949-bib-0036] Raunsgard, A. , Opedal, Ø. H. , Ekrem, R. K. , Wright, J. , Bolstad, G. H. , Armbruster, W. S. , & Pélabon, C. (2018). Intersexual conflict over seed size is stronger in more outcrossed populations of a mixed‐mating plant. Proceedings of the National Academy of Sciences of the United States of America, 115, 11561–11566. 10.1073/pnas.1810979115 30282740 PMC6233115

[jeb13949-bib-0037] Revell, L. J. (2012). phytools: An R package for phylogenetic comparative biology (and other things). Methods in Ecology and Evolution, 3, 217–223. 10.1111/j.2041-210X.2011.00169.x

[jeb13949-bib-0038] Robertson, K. , Goldberg, E. E. , & Igić, B. (2011). Comparative evidence for the correlated evolution of polyploidy and self‐compatibility in Solanaceae. Evolution, 65, 139–155. 10.1111/j.1558-5646.2010.01099.x 20722729

[jeb13949-bib-0039] Sharma, N. , Koul, A. K. , & Kaul, V. (1999). Pattern of resource allocation of six *Plantago* species with different breeding systems. Journal of Plant Research, 112, 1–5. 10.1007/pl00013850

[jeb13949-bib-0040] Shimizu, K. K. , & Tsuchimatsu, T. (2015). Evolution of selfing: Recurrent patterns in molecular adaptation. Annual Review of Ecology, Evolution, and Systematics, 46, 593–622. 10.1146/annurev-ecolsys-112414-054249

[jeb13949-bib-0041] Sicard, A. , & Lenhard, M. (2011). The selfing syndrome: A model for studying the genetic and evolutionary basis of morphological adaptation in plants. Annals of Botany, 107, 1433–1443. 10.1093/aob/mcr023 21303786 PMC3108801

[jeb13949-bib-0042] Smith, C. C. , & Fretwell, S. D. (1974). The optimal balance between size and number of offspring. The American Naturalist, 108, 499–506. 10.1086/282929

[jeb13949-bib-0043] Stanton, M. L. (1984). Seed variation in wild radish: Effect of seed size on components of seedling and adult fitness. Ecology, 65, 1105–1112. 10.2307/1938318

[jeb13949-bib-0044] Stebbins, G. L. (1950). Variation and evolution in plants, variation and evolution in plants. Columbia University Press. 10.7312/steb94536

[jeb13949-bib-0024] Stebbins, G. L. (1974). Flowering plants: Evolution above the species level. Belknap Press of Harvard University Press.

[jeb13949-bib-0045] Stevens, A. V. , Nicotra, A. B. , Godfree, R. C. , & Guja, L. K. (2020). Polyploidy affects the seed, dormancy and seedling characteristics of a perennial grass, conferring an advantage in stressful climates. Plant Biology, 22, 500–513. 10.1111/plb.13094 32011086

[jeb13949-bib-0046] Tamme, R. , Götzenberger, L. , Zobel, M. , Bullock, J. M. , Hooftman, D. A. P. , Kaasik, A. , & Pärtel, M. (2014). Predicting species’ maximum dispersal distances from simple plant traits. Ecology, 95, 505–513. 10.1890/13-1000.1 24669743

[jeb13949-bib-0047] Tsuchimatsu, T. , Kaiser, P. , Yew, C.‐L. , Bachelier, J. B. , & Shimizu, K. K. (2012). Recent loss of self‐incompatibility by degradation of the male component in Allotetraploid *Arabidopsis kamchatica* . PLOS Genetics, 8, e1002838. 10.1371/journal.pgen.1002838 22844253 PMC3405996

[jeb13949-bib-0048] Tsuchimatsu, T. , Kakui, H. , Yamazaki, M. , Marona, C. , Tsutsui, H. , Hedhly, A. , Meng, D. , Sato, Y. , Städler, T. , Grossniklaus, U. , Kanaoka, M. M. , Lenhard, M. , Nordborg, M. , & Shimizu, K. K. (2020). Adaptive reduction of male gamete number in the selfing plant *Arabidopsis thaliana* . Nature Communications, 11, 2885. 10.1038/s41467-020-16679-7 PMC7280297 32514036

[jeb13949-bib-0049] Webb, C. O. , & Donoghue, M. J. (2005). Phylomatic: Tree assembly for applied phylogenetics. Molecular Ecology Notes, 5, 181–183. 10.1111/j.1471-8286.2004.00829.x

[jeb13949-bib-0050] Willi, Y. (2013). The battle of the sexes over seed size: Support for both kinship genomic imprinting and interlocus contest evolution. American Naturalist, 181, 787–798. 10.1086/670196 23669541

[jeb13949-bib-0051] Woźniak, N. J. , Kappel, C. , Marona, C. , Altschmied, L. , Neuffer, B. , & Sicard, A. (2020). A similar genetic architecture underlies the convergent evolution of the selfing syndrome in *Capsella* . The Plant Cell, 32, 935–949. 10.1105/TPC.19.00551 31964802 PMC7145481

[jeb13949-bib-0052] Zanne, A. E. , Tank, D. C. , Cornwell, W. K. , Eastman, J. M. , Smith, S. A. , FitzJohn, R. G. , McGlinn, D. J. , O’Meara, B. C. , Moles, A. T. , Reich, P. B. , Royer, D. L. , Soltis, D. E. , Stevens, P. F. , Westoby, M. , Wright, I. J. , Aarssen, L. , Bertin, R. I. , Calaminus, A. , Govaerts, R. , … Beaulieu, J. M. (2014). Three keys to the radiation of angiosperms into freezing environments. Nature, 506, 89–92. 10.1038/nature12872 24362564

