# Supplementary material for: Evolution of seed mass associated with mating systems in multiple plant families
Source: J Evol Biol. 2021 Oct 26;34(12):1981–7. doi: 10.1111/jeb.13949 (PMC9298147; doi:10.1111/jeb.13949)
Supplement: Supplementary file 1 — Fig S1 [file JEB-34-1981-s001.docx]

Supplementary Fig. 1. Genus-level phylogenetic trees of Asteraceae (A), Brassicaceae (B), and Solanaceae (C) generated from phylomatic (Webb & Donoghue, 2005). Trees were drawn by using R package “ape” (Paradis & Schliep, 2019).
